# Design data for the 3D printer modification to print gels and pastes and the corresponding firmware

**DOI:** 10.1016/j.dib.2021.106974

**Published:** 2021-03-21

**Authors:** Saumil Sudhir Vadodaria, Eleanor Warner, Ian Norton, Tom B. Mills

**Affiliations:** School of Chemical Engineering, University of Birmingham, Edgbaston Campus, Birmingham, B15 2TT, UK

**Keywords:** 3D Printing, Additive manufacturing, Hydrogels, Pastes, Syringe pump, Customisation, Modification, Firmware

## Abstract

In order to deposit gel and paste-like materials, a commercially available HICTOP Prusa i3 plastic 3D printer was modified. The modification included replacing the existing plastic microextruder with a customised 3D printed syringe pump which could hold a syringe containing the printing material. The arrangement also allowed the temperature in the syringe to be controlled. Since the hardware of the printer was changed significantly, a new firmware was loaded on the 3D printer which was customised to enable it to perform its new function.

The present data consists of the 3D image files of the syringe pump assembly and instructions on how to assemble the components. It also provides a copy of the modified firmware with a list of the changes made to it.

This data will allow the readers to modify a similar type of 3D printer to print pastes and gels. This can be achieved by recreating the entire syringe pump assembly by 3D printing the given 3D image file data. With some changes, these designs can also be adapted to a variety of different printers. Similarly, the given firmware can also be loaded onto a similar type of printer. The list and explanation of the changes made to the firmware also allow such changes to be made to the respective firmwares of a variety of different printers.

## Specifications Table

**Table:** 

Subject	Materials Science
Specific subject area	Materials Processing. These data describe how a plastic 3D printer was modified in terms of hardware and firmware to make it deposit paste and gel materials.
Type of data	Images – 3D Figures Marlin Firmware (Opens in Arduino software)
How data were acquired	The 3D images of syringe pump components were produced using a web-based design software called Tinkercad. The Marlin firmware was open-source and was downloaded from the Internet (more details have been given in the main text). It was then modified in a software called Arduino.
Data format	Original designs Modified open-source code
Parameters for data collection	The 3D designs were made in such a way that the assembled pump fits the 3D printer as well as the syringe. The firmware was changed to ensure that the extruder parameters were suitable for pushing a plunger in the syringe rather than pushing a plastic filament into the microextruder. The thermal settings were also changed to heat the syringe to a much lower temperature compared to a microextruder.
Description of data collection	The dimensions of the syringe, the 3D printer and the stepper motor were measured and were taken into consideration when designing the syringe pump.
Data source location	School of Chemical Engineering, University of Birmingham Birmingham United Kingdom 52°28;48′′N 01°54′09′′
Data accessibility	Repository name: Mendeley Data Data identification number: https://doi.org/10.17632/4pmjj3dbvr.1 Direct URL to data: https://data.mendeley.com/datasets/4pmjj3dbvr/draft?a=08e8f449=3775=47fa-a223-5f498a328322
Related research article	S. S. Vadodaria, E. Warner, I. Norton and T. B. Mills, Thermoreversible Gels – Optimisation of Processing Parameters in Fused Deposition Modelling, Colloids and Surfaces A: Physicochemical and Engineering Aspects, Under Revision. doi:10.1016/j.colsurfa.2021.126399 https://www.sciencedirect.com/science/article/abs/pii/S0927775721002685

## Value of the Data

• These data could be used to modify a HICTOP Prusa i3 3D printer to deposit gel or paste-like materials by incorporating a syringe pump into it.
• Researchers working on 3D printing of soft matter formulations (pastes or gels) for food, healthcare or other applications can use these data as a guide to modify their 3D printers.
• In order to modify a HICTOP Prusa i3 3D printer, the 3D images provided in the data need to be printed and the parts can be assembled with the printer. The provided firmware can be loaded directly onto the printer. For other printers, the 3D images need some alterations and the corresponding firmware has to be amended as per the instruction provided in this article.

## 1 Data Description

The dataset is divided into two components: (i) 3D images of the syringe pump with .stl extension and (ii) Firmware files. The images in .stl files can be viewed/edited by most computer aided design (CAD) software packages. Objects (the parts of the syringe pumps) can be fabricated based on these files using 3D printers. The firmware files can be read/edited using a software called Arduino which can be obtained from www.arduino.cc. This software package can also be used to load the firmware into the mainboard of a compatible 3D printer.

The list of included files has been provided in Table 1:

**Table 1:** The list of all data files, including the 3D images and the firmware.

	File name	Description
**(1)**	Syringe Pump Main Body.stl	Contains the image of the parts of the syringe pump which are attached in a single structure. These parts include a plate with screws which attach the entire assembly to the printer, the mount for the stepper motor which drives the extrusion as well as the ‘barrel plate’ which is designed to closely fit the fins and the barrel of the metallic syringe that was used in the study.
**(2)**	Nut Plate.stl	The image in this file contains a stand-alone plate in which a leadscrew nut can be incorporated. This nut moves in linear direction in response to the rotational movement of the leadscrew which passes through the nut, moving the entire plate with itself. This plate also carries the end of the syringe plunger which moves linearly (i.e. in forward or backward directions) with the plate when the plate itself moves as a result of the leadscrew nut movement.
**(3)**	Nut Plate Lid.stl	This file contains the image of the so-called ‘nut plate lid’ which is used to secure the end of the syringe plunger in the nut plate. It has been designed to closely fit the plunger.
**(4)**	Barrel Plate Lid.stl	Consists of the lid for the ‘barrel plate’ which is the part of the main body. Putting this lid on the barrel plate secures the barrel and its fins to the syringe pump body.
**(5)**	Rod Aligner.stl	The component shown in this image file is an optional accessory which needs to be used to align the two ‘guide rods’ and the leadscrew in a single line, if required. This makes the syringe pump operation smoother.
**(6)**	Firmware.zip	This file is a collection of codes that can be viewed/edited in Arduino package and be loaded into a compatible 3D printer.

## 2 Experimental Design, Materials and Methods

### 2.1 Syringe pump assembly

An open-source Prusa i3 HICTOP 3D printer was acquired in form of an assembly kit. It was assembled and was found to be functioning properly upon test printing of a plastic specimen. Subsequently, its hotend extruder assembly was dismantled in order to modify the printer.

The prime consideration about converting the plastic extruder in a 3D printer to a syringe pump is of the stepper motor. The stepper motor in a plastic extruder loads/removes the plastic filament inside the hotend. The same stepper motor needs to be repurposed to push a plunger into a syringe or to retract it. This could be achieved by the use of a leadscrew, which translates a rotational motion (by the stepper motor) into a linear motion (by its nut moving across the leadscrew corresponding to the rotation).

A leadscrew with 0.5 mm lead (the axial distance corresponding to one full rotation) and 5 mm diameter was attached to the stepper motor using a flex coupling. Both leadscrew and the coupling were acquired from Reliance Precision Limited, UK.

**Fig. 1:**
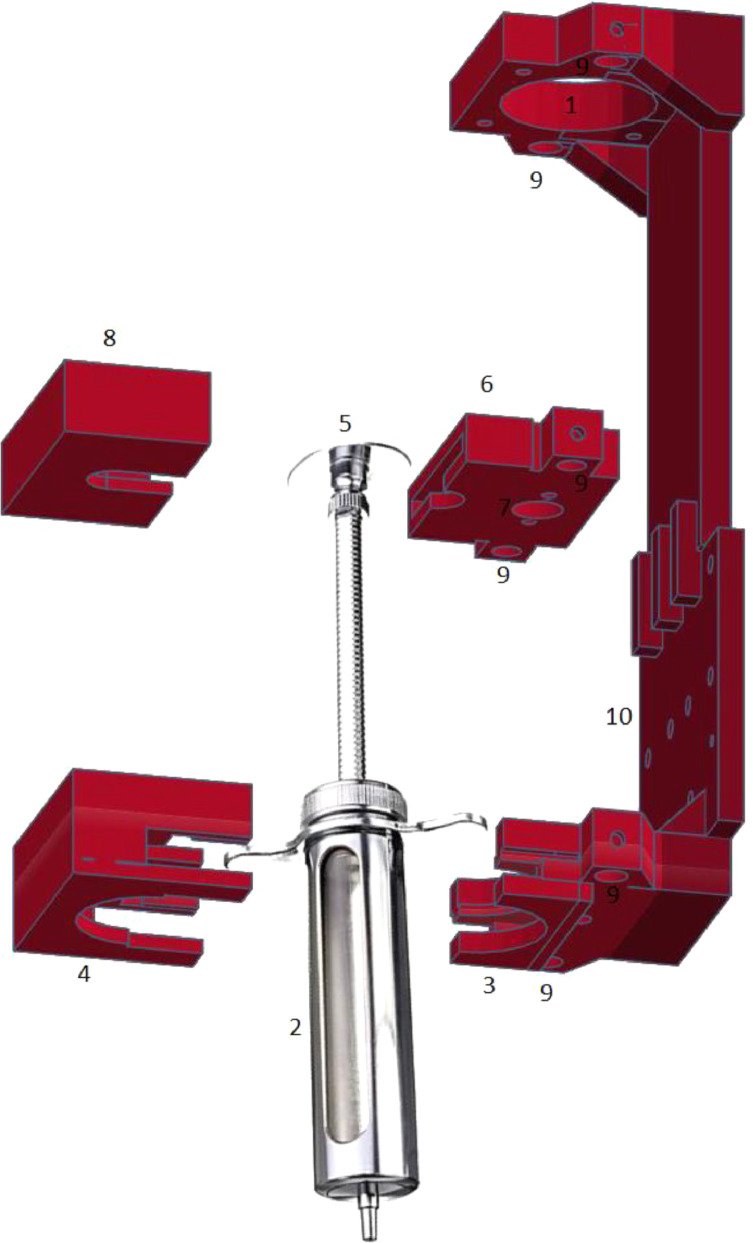
The exploded view of the syringe pump assembly along with the metallic syringe. The numbered components have been described in the main text.

A 30 ml metallic veterinary syringe was acquired from Jiashan Veterinary Equipment, China. A metallic syringe was chosen because of its better thermal conductivity compared to a plastic syringe. The dimensions of this syringe were measured, and an assembly of syringe pump was designed in an online software called Tinkercad (www.tinkercad.com) in such a way that could closely fit both the syringe and the 3D printer setup. This kit was then 3D printed and was assembled together. Various components of the syringe pump are shown in the exploded view of the assembly in Fig. 1.

The working principle behind the syringe pump can be described as follows:

The stepper motor is mounted on the very top of the assembly, with the coupling and the leadscrew connected to it through the hole (1) in Fig. 1. The syringe barrel (2) is firmly held by the barrel plate (3) in a static, vertical position and secured by the barrel plate lid (4). The syringe plunger (5) is held in a stand-alone nut plate (6) which also houses the leadscrew nut in a hole (7). The syringe plunger is secured in the nut plate using the nut plate lid (8). The movement of motor displaces the leadscrew nut and the plunger in an axial position, enabling extrusion and retraction. Two parallel vertical axes exist in the syringe pump, one passing through the centre of plunger and barrel, and the other corresponding to the motor shaft, coupling and the leadscrew. The integrity and the parallelism of both these axes are maintained by the so-called ‘guide-rods’ which are inserted in several holes (9). These rods provide additional strength to the pump and resist any deformation induced by the extrusion of firm materials.

**Fig. 2:**
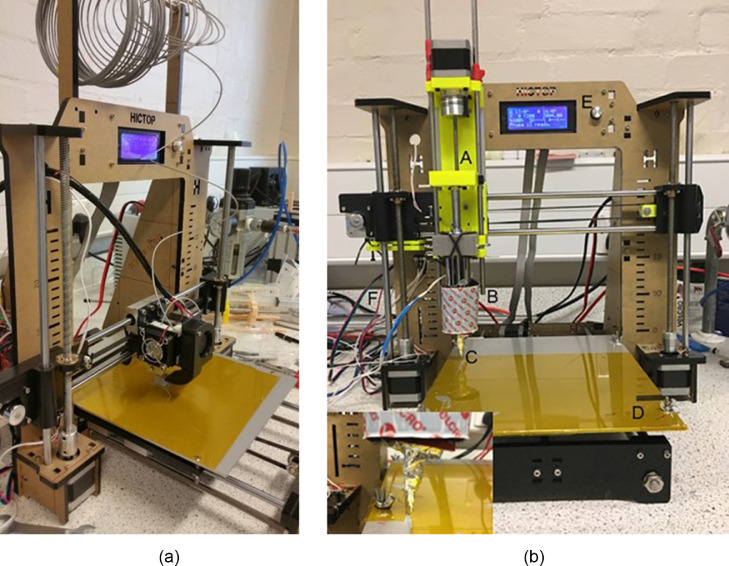
HICTOP Prusa i3 plastic 3D printer (a) before and (b) after the modification.

This entire assembly is attached to the printer using the screw plate (10). This plate was designed considering the screw positions in the printer where the plastic extruder was previously attached. This plate needs to be re-designed when working with a printer that may have different screw positions. Figure 2 (a) and (b) show the printer prior to and after the modification respectively.

### 2.2 Firmware

The firmware was downloaded from www.reprap.pt and was referred to as ‘Prusa i3 Rework Firmware’ on www.reprap.org. It was then viewed and edited in Arduino software package (Marlin.ino file was opened). All the changes were made in the Configuration.h tab. The summary of changes is given in Table 2.

**Table 2:** The summary of firmware changes.

	Line numbers	Code	Explanation
1	Line 27	#define BAUDRATE 115200	The baudrate needs to be adjusted to a value which allows the computer to communicate with the 3D printer.
2	Line 75	#define MOTHERBOARD 33	RAMPS 1.3/1.4 board is represented by the number 33. A list of boards and their numbers has been provided in the firmware, above Line 75.
3	Line 86	#define EXTRUDERS 1	Only one extruder is being used.
4	Line 174	#define BANG_MAX 50	The default value is 255 which corresponds to the full current being drawn by the heater, leading to high temperatures. Since the desirable temperatures for syringe are significantly lower, a value of 50 is more appropriate as it draws much lower current.
5	Line 175	#define PID_MAX 1	Similar to Line 174.
6	Line 181	#define PID_INTEGRAL_DRIVE_MAX 50	Similar to Line 175.
7	Lines 207—209	#define DEFAULT_Kp 23.05 #define DEFAULT_Ki 2.00 #define DEFAULT_Kd 66.47	Related to the algorithm for an effective temperature control.
8	Line 231	#define MAX_BED_POWER 128	Similar to Line 174 but for the bed heater. The bed heater was repurposed to provide heating near the nozzle.
9	Line 319	#define INVERT_E0_DIR true	For ‘direct drive’ type extrusion.

The firmware was then loaded into the 3D printer. In order to perform printing, an open-source software called ‘Repetier’ was downloaded and used. In order to calibrate the syringe pump to extrude the correct quantity, the so-called ‘extrusion multiplier factor’ from Repetier was optimised as per the requirements. This design of syringe pump and the firmware were also used in a previous study [1], whereas those in another study were heavily inspired from it [2].

## Ethics Statement

The work did not involve any experiments on human or animal subjects. None of the data was collected from any social media platforms.

## Funding Source

This research was funded by the Engineering and Physical Sciences Research Council (EP/K030957/1).

## CRediT Author Statement

**Saumil Sudhir Vadodaria:** Writing - original draft, Writing - review & editing, Software, Validation; **Eleanor Warner:** Methodology, Investigation, Visualization; **Ian Norton:** Funding acquisition; **Tom B. Mills:** Conceptualization, Supervision.

## Declaration of Competing Interest

The authors declare that they have no known competing financial interests or personal relationships which have or could be perceived to have influenced the work reported in this article.
