# Corrigendum to design data for the 3D printer modification to print gels and pastes and the corresponding firmware

**DOI:** 10.1016/j.dib.2021.107251

**Published:** 2021-06-28

**Authors:** Saumil Sudhir Vadodaria, Eleanor Warner, Ian Norton, Tom B. Mills

**Affiliations:** School of Chemical Engineering, University of Birmingham, Edgbaston Campus, Birmingham, B15 2TT, UK

The authors regret that:

The EPSRC grant number currently stated in Funding Source section is `EP/K030957/1', which is incorrect. The correct funding source is `EP/N024818/1'.

The authors would like to apologise for any inconvenience caused.

